# Metastases to the breast: great radiological mimicker of primary breast carcinoma and a forgotten entity. A case series of three patients and a review of the literature

**DOI:** 10.1259/bjrcr.20160137

**Published:** 2017-03-14

**Authors:** Jaspreet Sangha Brar, Lena Lo, Jill Wong

**Affiliations:** Department of Oncologic Imaging, National Cancer Centre, Singapore, Singapore

## Abstract

Metastases to breast accounts for 0.5–1.3% of all breast malignancies, with the exclusion of leukaemia and lymphoma. These have a wide range of clinical and radiological manifestations and their diagnosis is difficult. There is a need to distinguish them from primary breast carcinoma to prevent unnecessary mastectomy. Imaging and immunohistological correlation plays a vital role in distinguishing this. Our case series review describes the clinical presentation, radiological and histopathological appearances of three patients who presented to our institution.

## Methods

Retrospective review of three patients with metastatic breast lesions was carried out using the images on PACS and other hospital records. 

## Results

The various radiological appearances of breast lesions of these three patients were reviewed and these were histological proven metastases from lung (n=3). Out of these three patients, only one had a history of malignancy. Two out of three patients did not have any breast complaints and the lesion was detected on routine screening. Two patients did not have any abnormality on mammogram and the abnormality was found on further additional imaging such as ultrasonography (US), CT and MRI. We also found that these tumours were aggressive with widespread systemic metastases at the time of presentation. Accurate differentiation from primary carcinoma is important because the treatment and prognosis differ significantly.

## Case 1

A 66-year-old Chinese lady presented with a retracted nipple and generalised thickening over the lateral aspect of her right breast. She had a medical history of metastatic adenocarcinoma of the lung which was initially diagnosed approximately one and a half years prior to the development of her breast symptoms.

On clinical examination, no palpable mass was found in both breasts, but there was mild skin thickening in right lateral breast. Mammography showed bilateral dense breasts with no dominant mass, suspicious micro-calcification or architectural distortion.

**Figure 1.**
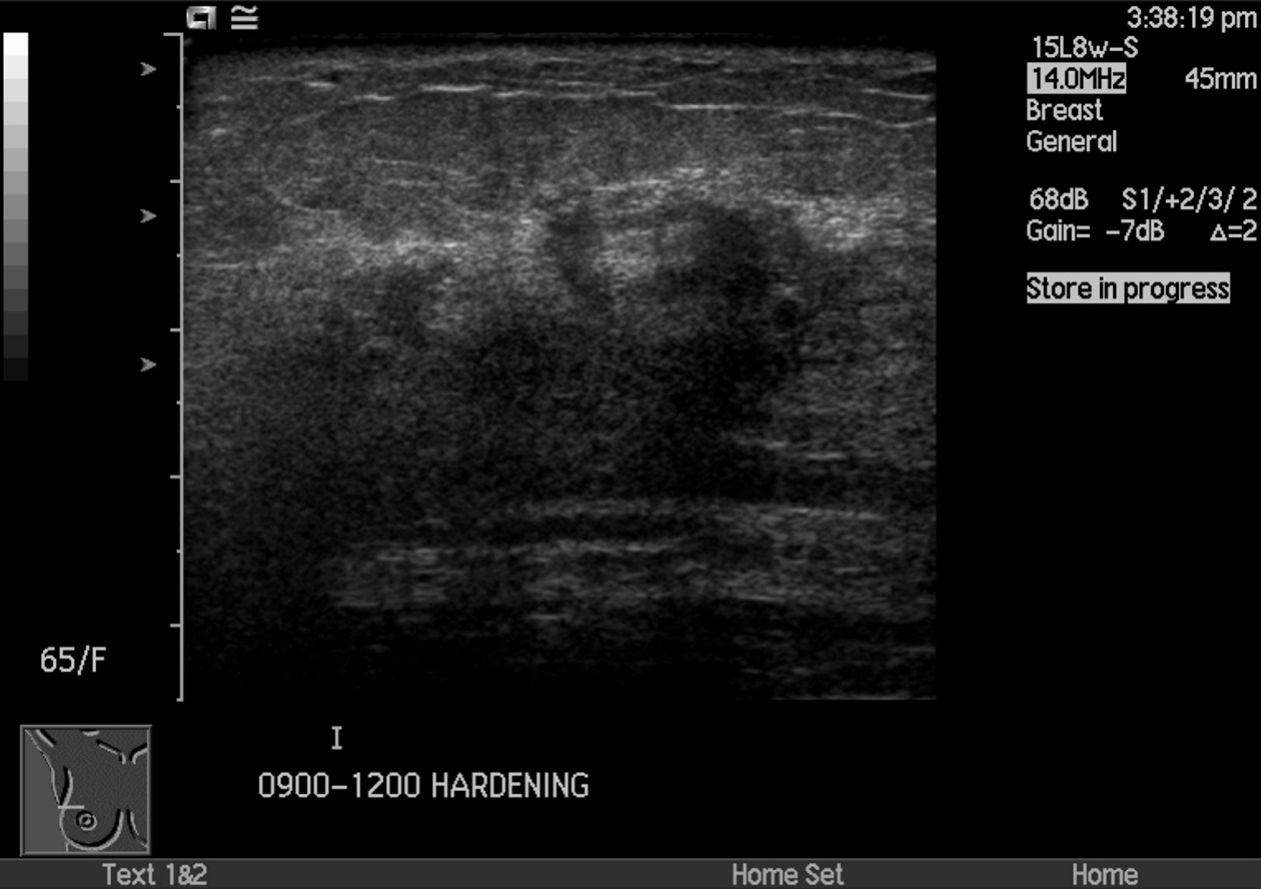
US scan showed diffuse stromal thickening in the background of heterogeneous parenchyma corresponding to the area of palpable thickening in the right lateral breast.

**Figure 2.**
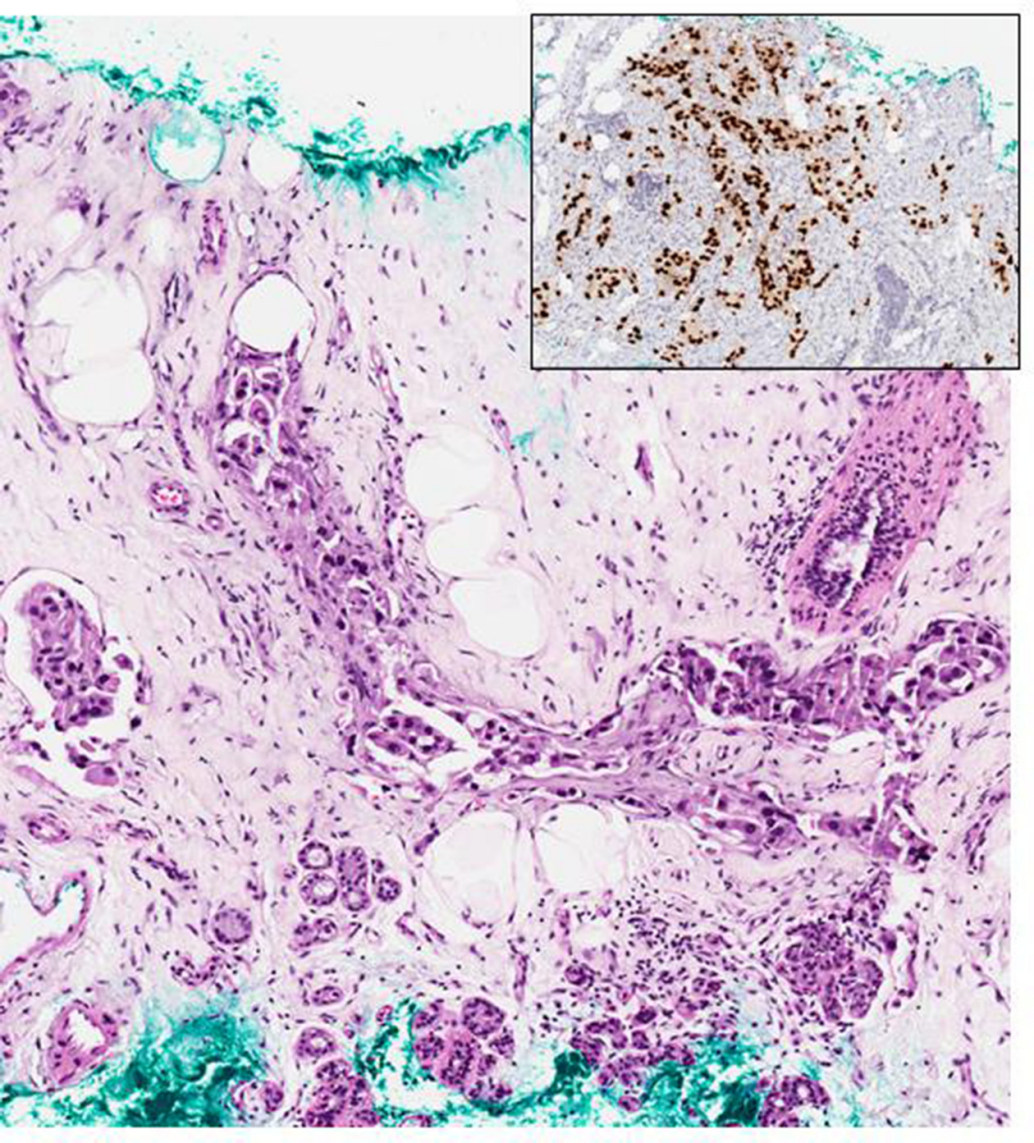
Tru-cut biopsy of breast lump showed invasive carcinoma, with diffusely positive staining for TTF-1 (inset) on immunohistochemistry (TTF-1: Thyroid transcription factor-1).

Ultrasonography, however, revealed diffuse stromal thickening in the background of heterogeneous parenchyma corresponding to the area of palpable thickening in the right lateral breast with no focal mass or nodule (Figure 1). Ultrasound-guided 14G core needle biopsy was performed, and histology confirmed a metastatic adenocarcinoma with an immune-profile favouring a lung primary (Figure 2).

**Figure 3.**
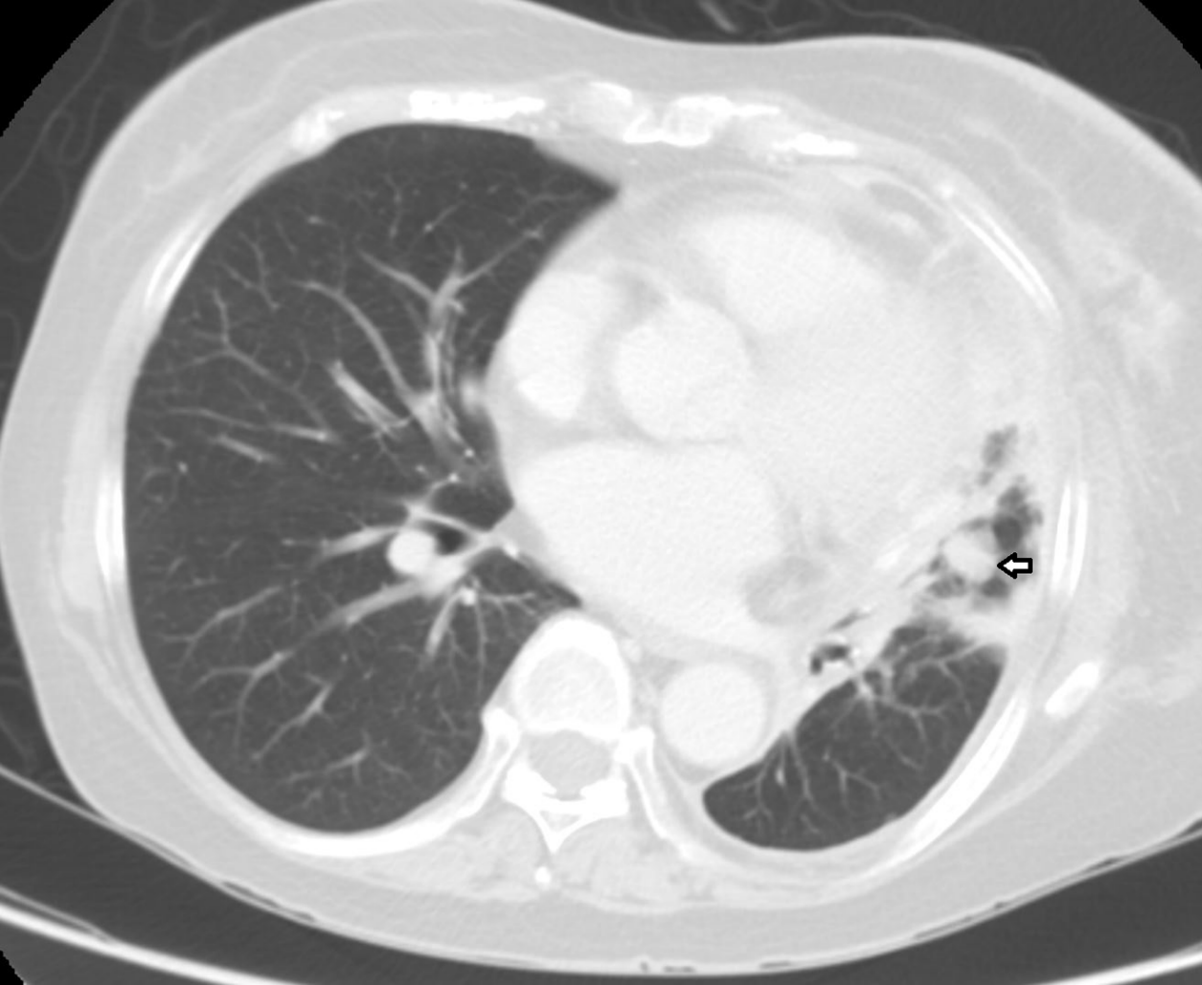
CT scan in the lung window showed a mass in the lingula lobe of the lung, in keeping with a primary tumour.

**Figure 4.**
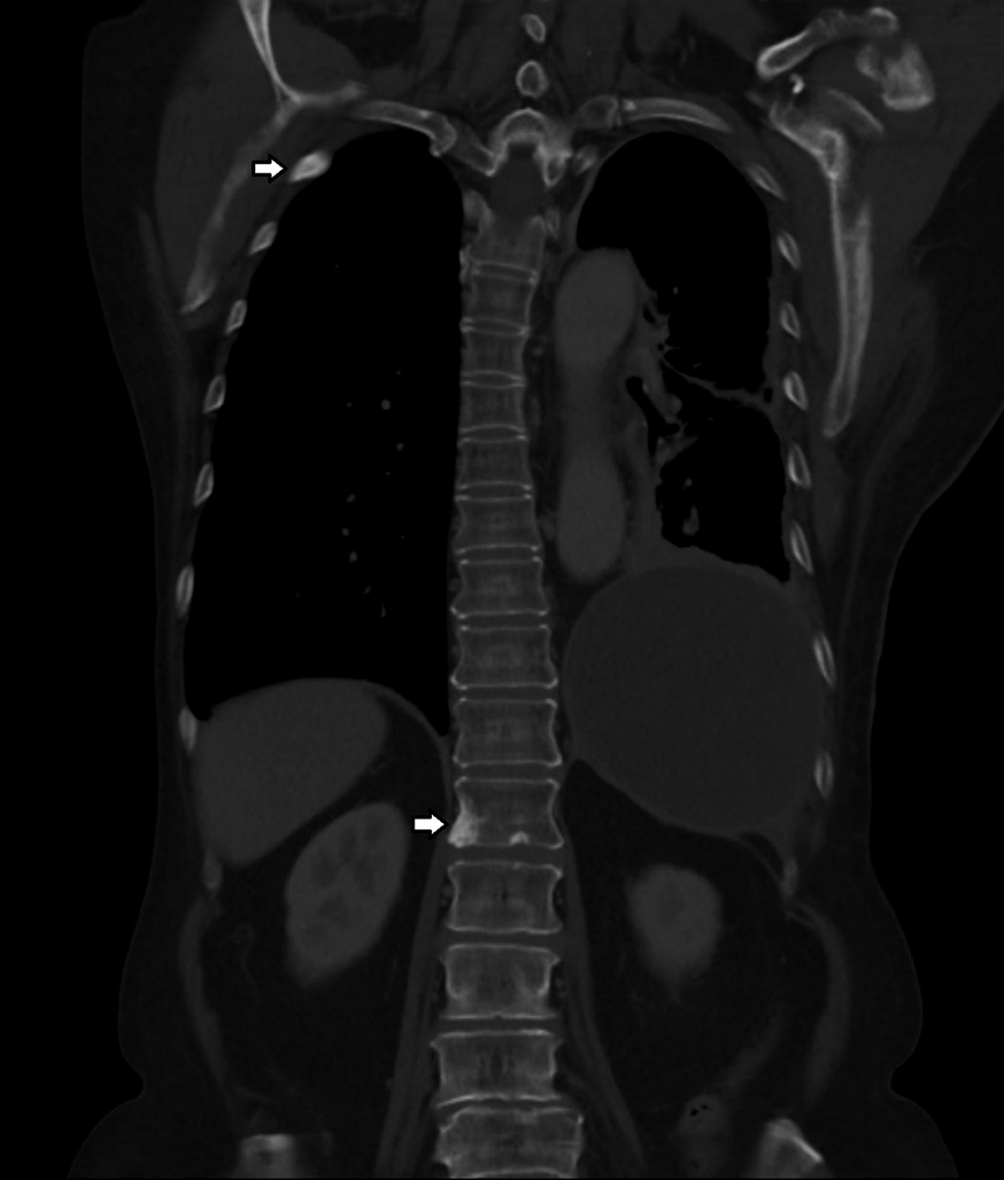
CT scan in the bone window showed sclerotic bony metastases to the ribs and spine.

Further imaging with a CT scan showed a lingula lobe primary tumour with extensive skeletal metastasis (Figures 3 and 4). Shortly thereafter, she progressed on to develop metastases to the cerebrospinal fluid (CSF) and succumbed within a month from diagnosis.

## Case 2

A 59-year-old Chinese lady initially presented to her family physician for a routine check-up. Routine blood screening showed raised levels of tumour markers with abnormally raised levels CEA, CA125, CA153 and CA19.9. She had a previous total hysterectomy and bilateral salpingo-oophorectomy 15 years ago, for adenomyosis. Apart from that, she had no history of malignancy.

**Figure 5.**
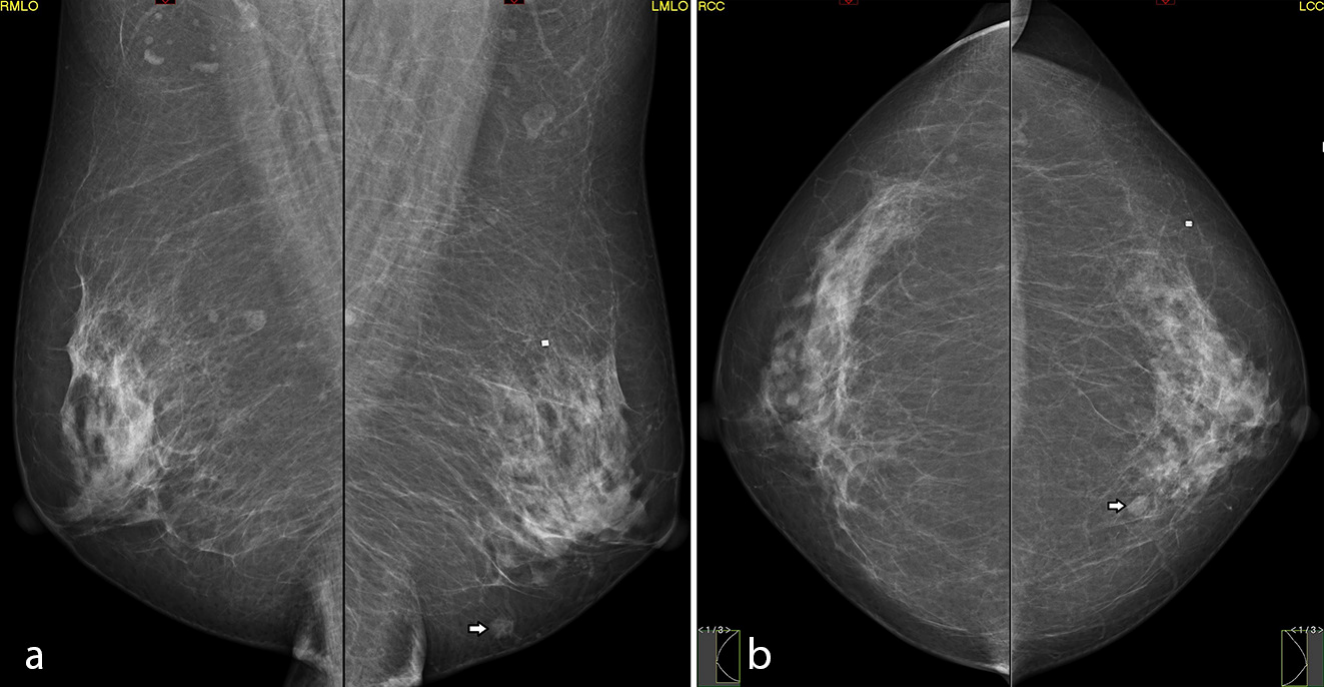
(a, b) Bilateral breast mammogram showed a 0.6 cm mass in the lower inner quadrant of the left breast.

During her consultation with the oncologist, she was found to have a mobile breast lump in the left breast. Subsequent mammography revealed a nodule in the lower inner quadrant of left breast with no associated micro-calcification or architectural distortion (Figure 5a,b).

**Figure 6.**
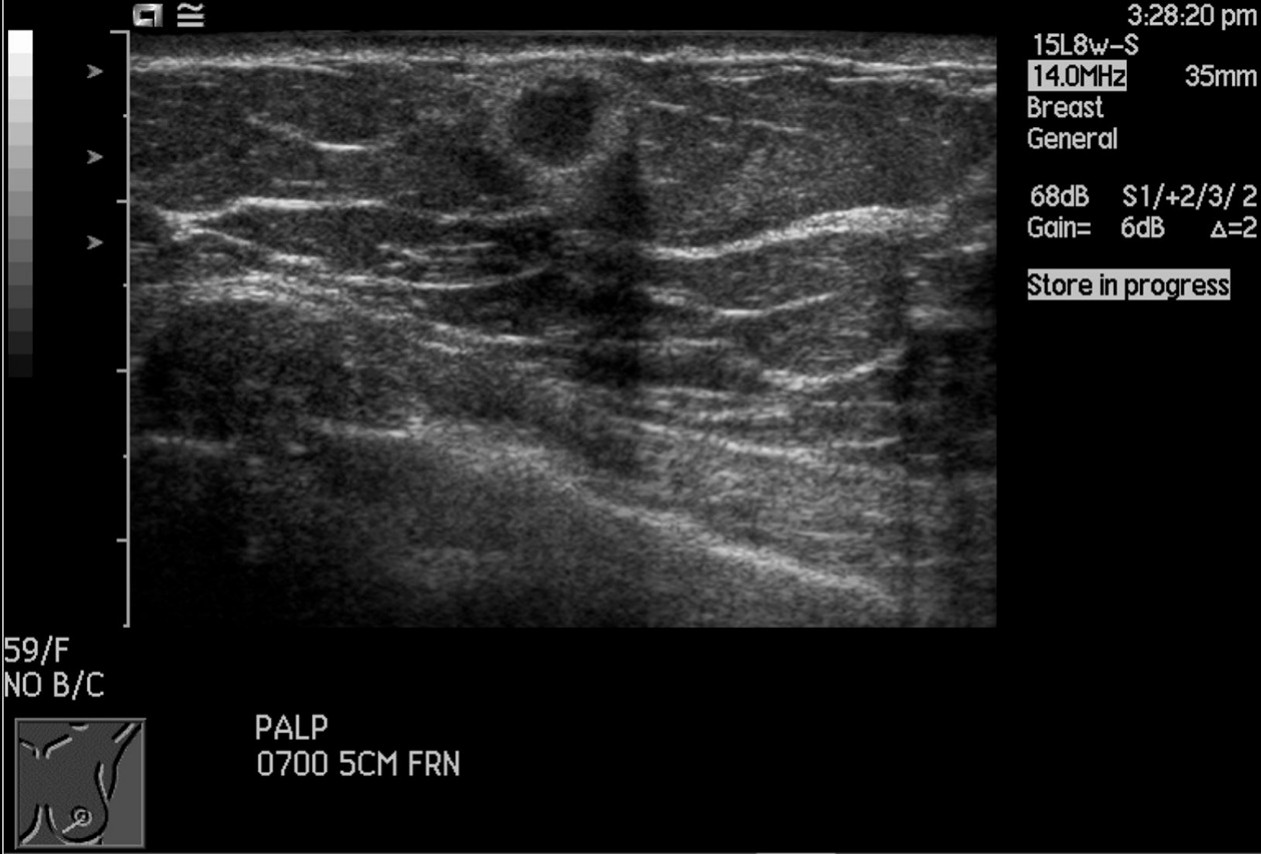
US scan showed an iso-echoic nodule at 7 o’clock in the left breast, corresponding to the mammographic lesion.

**Figure 7.**
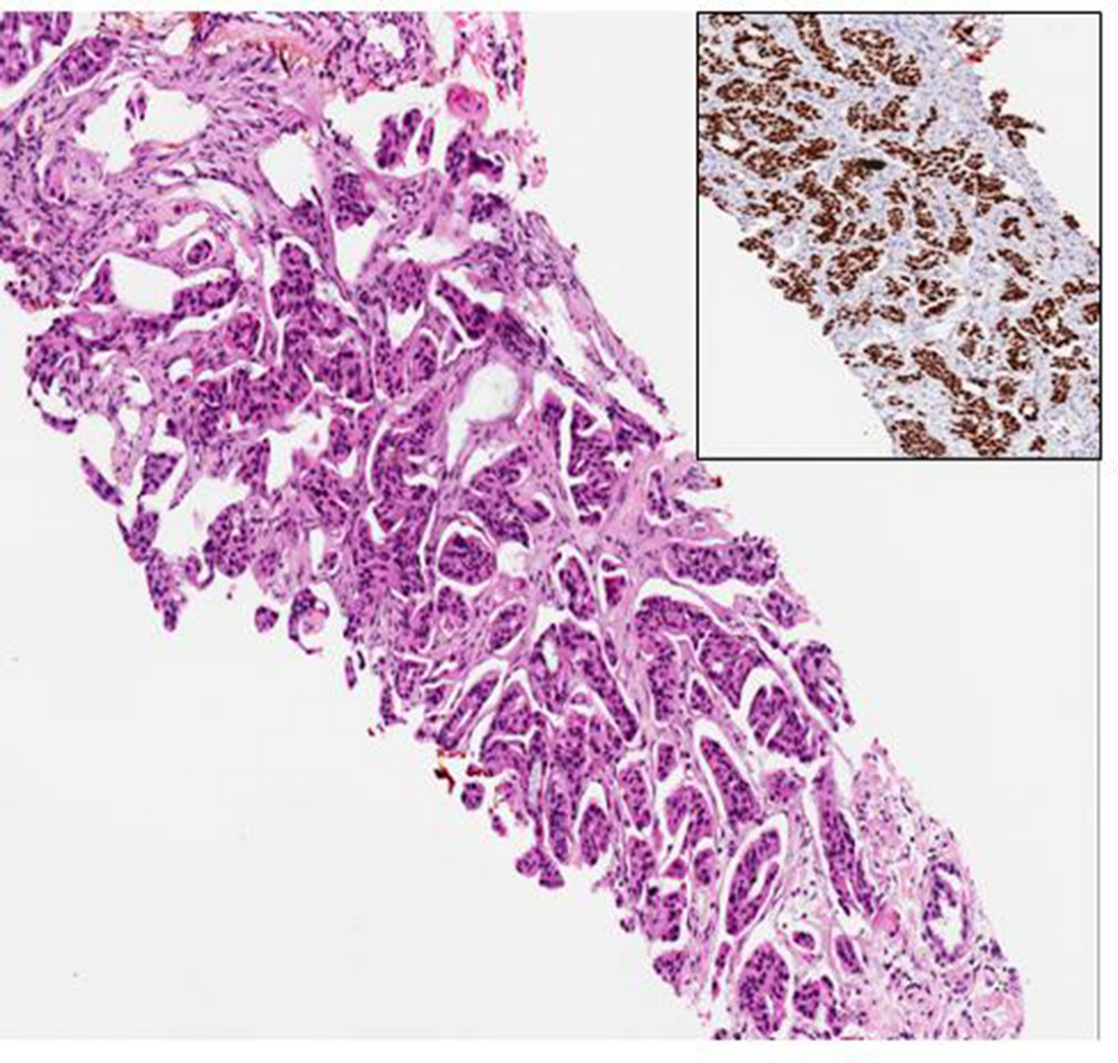
Tru-cut biopsy of breast lump showed extensive lymphovascular involvement by metastatic lung carcinoma. Inset: TTF-1 positive (thyroid transcription factor immunohistochemistry); 10× magnification.

Ultrasonography revealed a 0.6 × 0.6-cm iso-echoic nodule corresponding to the nodule in mammogram (Figure 6). Ultrasound-guided 14G core needle biopsy was performed, and histology with immunostaining revealed a metastatic adenocarcinoma from a lung primary (Figure 7).

A CT scan was done as part of her metastatic workup and it revealed a mass in right lower lobe, associated precarinal lymphadenopathy and bony metastases. The patient died approximately 2 months after diagnosis.

## Case 3

**Figure 8.**
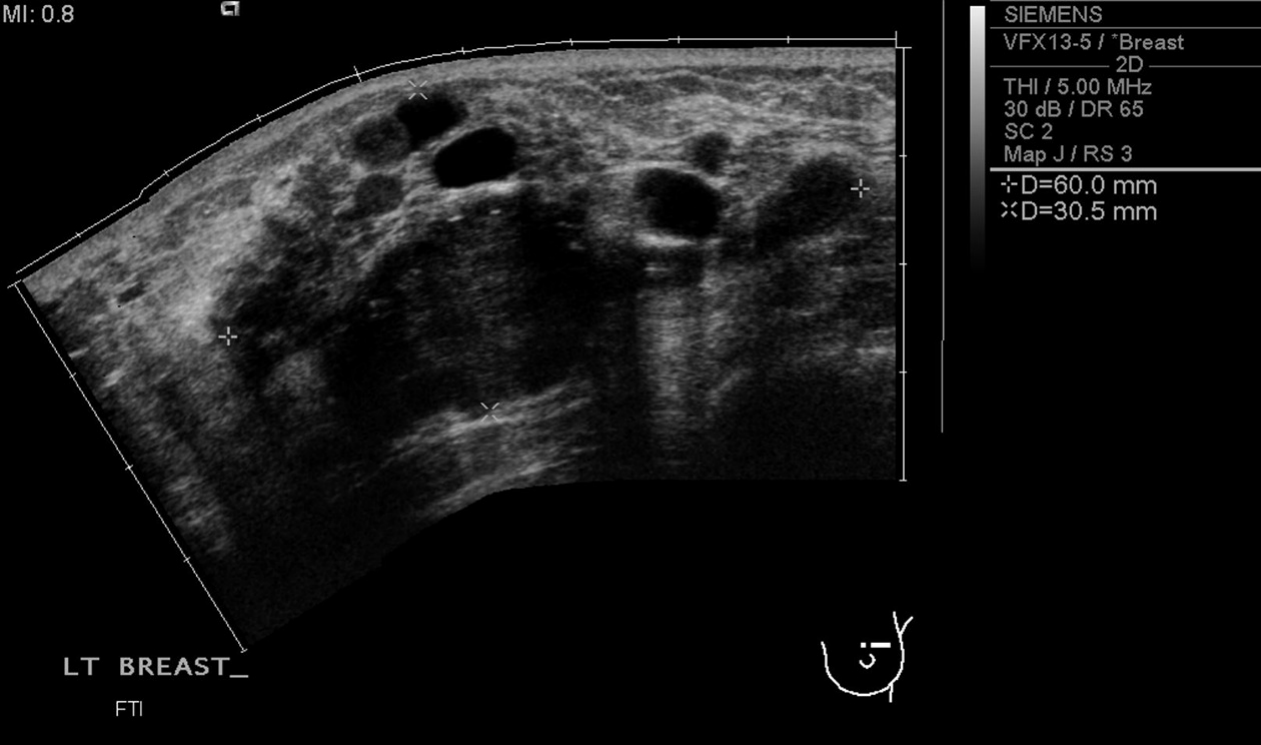
US scan showed a large lobulated mass at 12 o’clock in the left breast, surrounded by a few breast cysts.

A 40-year-old Chinese lady presented to our institution with a left breast mass. Mammography and ultrasonography of the left breast. Imaging revealed a lobulated mass in the upper quadrant at 12 o’clock position with associated ipsilateral axillary lymphadenopathy. Several large cysts were also noted in both breasts (Figure 8).

**Figure 9.**
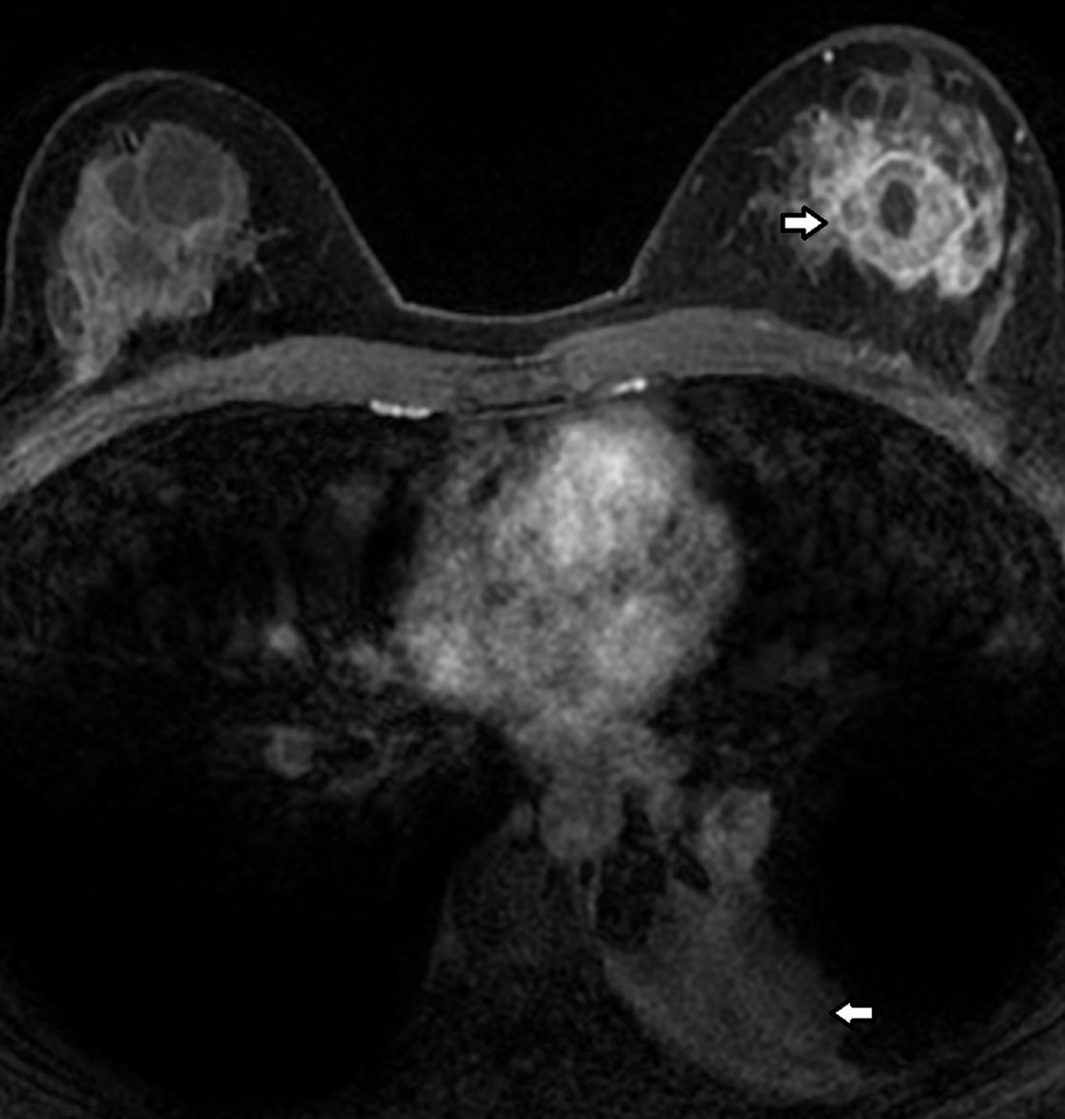
Post-contrast enhanced fat suppressed axial *T*_1_ weighted MR scan showed a left breast mass (upper arrow) and left lower lobe lung mass with associated atelectasis. (lower arrow).

**Figure 10.**
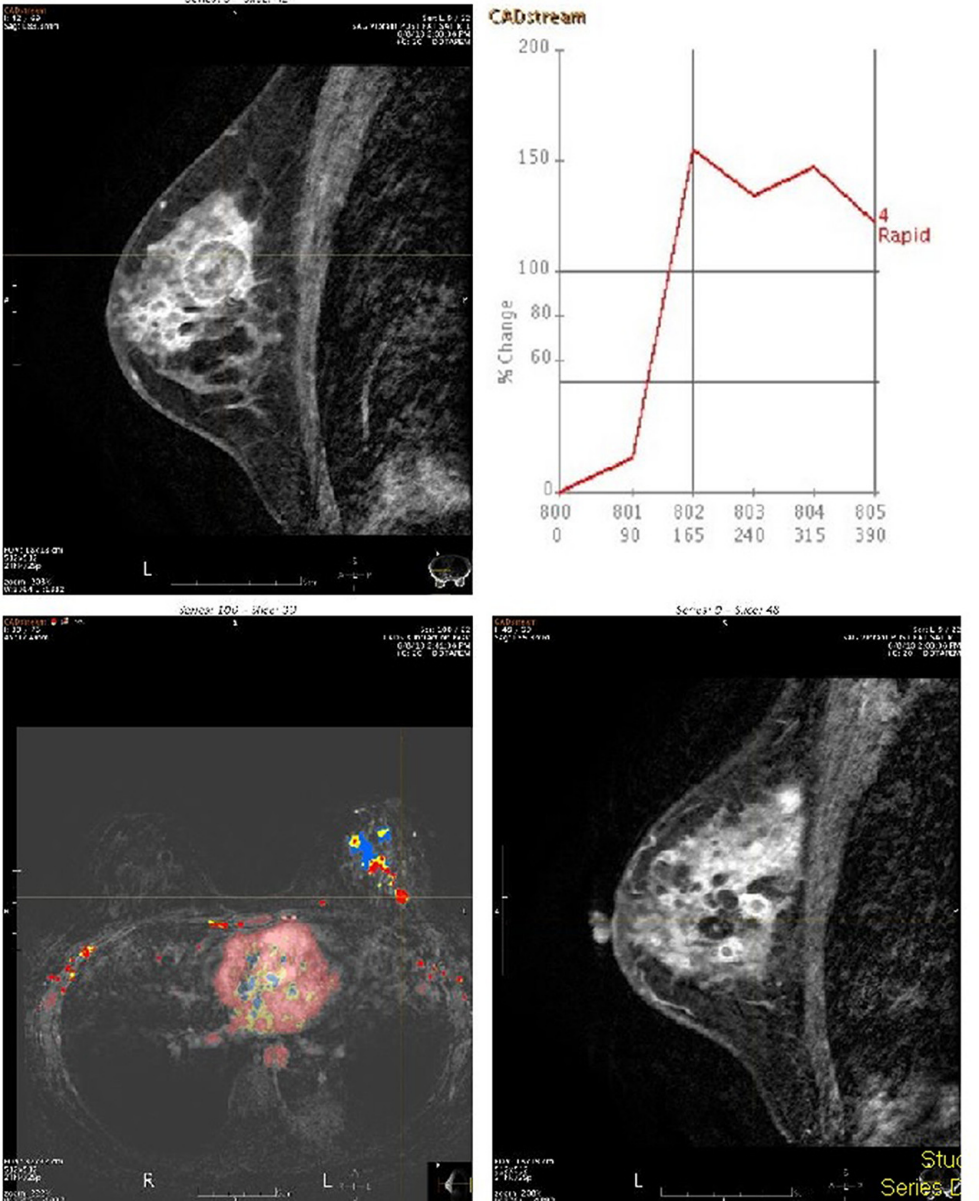
Post-contrast enhanced fat suppressed sagittal *T*_1_ weighted MR scan shows the dominant mass in the left breast with surrounding several smaller enhancing nodules compatible with metastatic lesions.. The image also shows the enhancement kinetic curve of the dominant lesion which is the “washout” or Type III pattern. This pattern is suggestive of malignancy.

**Figure 11.**
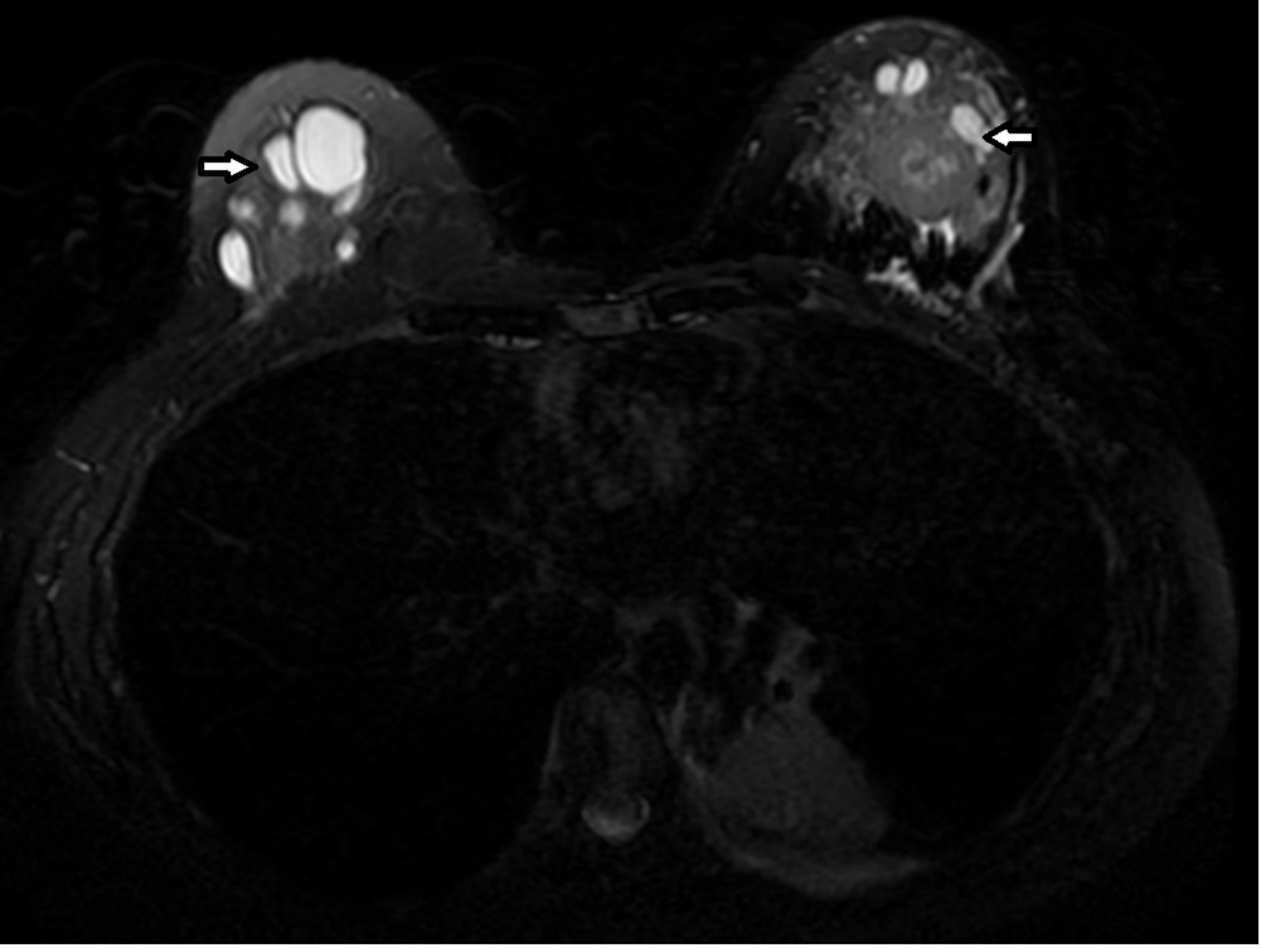
Unenhanced fat suppressed axial *T*_2_ weighted MR scan showed several cysts of varying sizes in both breasts.

MR of the breasts was performed to evaluate the size and number of lesions which were difficult to separate from the adjacent cysts. This showed a predominant 25  × 23 × 27-mm mass in the left breast, and several other smaller rim-enhancing lesions that demonstrated rapid enhancement with a plateau and washout on delayed phase. The smaller enhancing foci were thought to also represent metastatic lesions. The MR also further demonstrated a left lower lobe lung mass and associated atelectasis (Figures 9–11). On further questioning, the patient admitted to having had a cough for 1 month.

**Figure 12.**
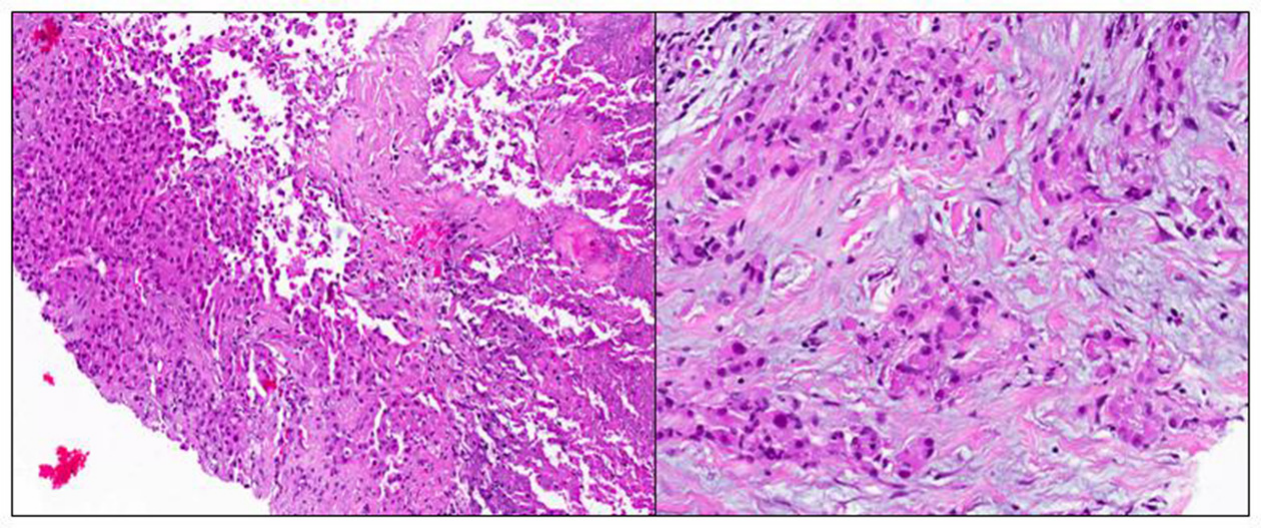
Tru-cut biopsy of breast lump showed a necrotic tumour with infiltrating pleomorphic cells that resemble a poorly differentiated invasive carcinoma, likely from the lung.

Ultrasound-guided 14G core biopsy of the left breast mass revealed a poorly differentiated adenocarcinoma, likely of lung origin (Figure 12). Fine-needle aspiration (FNA) of the left axillary nodes were also positive for malignant cells.

A staging CT scan confirmed the presence of a mass in the apical segment of the left lower lobe. There was also evidence of widespread sclerotic bony metastasis involving the axial skeleton. She was treated with chemotherapy but progressed to develop a solitary brain metastases in the left parietal lobe, approximately 13 months after diagnosis. Currently 7 years after diagnosis her disease remains fairly well controlled allowing her a good quality of life.

## Discussion

We report three patients with metastasis to the breast from pulmonary adenocarcinoma. Metastases to the breast are uncommon, but is a differential to consider in the workup of breast masses. The frequency reported in literature ranges from 0.5 to 1.3%, excluding leukaemia and lymphoma.^1–4^ Despite its rarity, metastatic disease to the breast is an important diagnostic clinical dilemma, because its treatment differs greatly from that of primary breast cancer. Sitzentfrey, in 1907, was the first to publish a case of ovarian carcinoma metastatic to the breast.^5^

According to the literature the most common primary tumours are melanomas and haematological malignancies followed by rare sites including lung, ovary, stomach, carcinomas of the liver, tonsil, pleura, pancreas, cervix, perineum, endometrium and bladder.^6–8^

Metastases to the breast can occur via two routes, namely the lymphatics and haematogenous, with different radiologic appearances. Lymphatic metastases tend to occur across the anterior chest wall to the opposite breast, mostly commonly from contralateral breast cancer. They can also rarely occur from stomach, ovarian and squamous carcinomas. The subcutaneous fat and glandular stroma become denser while the breast trabeculations become thicker. The overall appearance becomes indistinguishable from that of inflammatory breast cancer.^6^

Haematogenous breast metastases from extramammary malignancies are commonly located in the upper outer quadrant and are located superficially in subcutaneous tissue or immediately adjacent to the breast parenchyma that is relatively rich in blood supply.^6^ These are unlike primary breast carcinomas, which are usually deep seated as they arise from glandular parenchyma. Also, in the vast majority of metastases retraction of the skin or nipple is not demonstrated despite their superficial location.

Clinically, these patients tend to present with solitary discrete nodule or may be asymptomatic. Pain, tenderness or nipple discharge are unusual. On mammography, metastatic breast masses can be well circumscribed, ill circumscribed mass, an asymmetric density or occult. The lesions which were found in our series included a normal mammogram (n = 1) and well-circumscribed nodule/mass with no micro-calcification or architectural distortion (n = 2). Micro-calcifications are not usually associated with breast metastases. However, a few cases have been described in the literature from sites like ovary, thyroid and a few mucin producing gastrointestinal tract primary tumours.^4,6,7,9^ Typical US features of haematogeneous metastases include well circumscribed, single or multiple hypo echoic masses without spiculations, calcifications or architectural distortions. Posterior acoustic shadowing and desmoplastic reaction are also absent.

Based on our review of the existing literature, there has been no description of the MR appearances of metastases to the breast. One of the patients in our study had a breast MRI performed and it showed a rim-enhancement pattern with subsequent washout, which is suggestive of malignancy.

Overall, the clinical and radiological findings are indeterminate for a conclusive diagnosis, often mimicking a primary breast tumour. The well-defined appearance of these masses could pose difficulty in differentiating them from benign tumours such as fibro adenoma.^10^

Pre-surgical histopathological confirmation with the use of core needle biopsy is necessary in patients with breast lesions and especially in those with history of primary tumours in other organs, since the therapeutic options vary significantly in these two groups. These tumours often need the contribution of immunohistochemistry to confirm the diagnosis.

A metastatic carcinoma to the breast can histologically resemble a primary breast carcinoma, and hence immunohistochemistry should be performed to ascertain the ER, PR and Her2 status. The pathologist should also be informed of any clinical suspicion of metastasis or a previous primary carcinoma elsewhere so that additional immunostains may be performed. For example, TTF-1 and/or Napsin would point to a lung primary. p53 and WT-1 would point to an ovarian serous adenocarcinoma. Pax-2, CA-IX and RCC would point to a renal primary. S100, Melan-A and HMB45 point to a melanoma. A mucinous tumour, however, is generally not well supported by immunohistochemistry in terms of indicating the primary, as the immunostaining patterns overlap among the lung, breast, ovarian and GIT primaries. To further support breast carcinoma, GCDFP15, mammaglobin and ER, PR, Her2 are helpful.

## Learning points

1. The possibility of metastases to the breast should be considered in patients presenting with breast lumps who mayhave a history of other malignancy or present with other symptoms as well. Close radio-pathological correlation is necessary, as the therapeutic options and prognosis vary significantly in these two groups.
2. The imaging findings of metastatic breast lesions can be variable: ranging from being occult on mammogram to appearance of a benign looking nodule without suspicious features to having malignant features or an inflammatory looking pseudomass. Our experience is to have further specific immunostains performed on histopathology of these lesions to identify the origin of the tumour, in particular if a suspected other site of malignancy may be detected upon work up of the patient.

## Consent

Written informed consent for the case to be published (including images, case history and data) was obtained from the patient(s)/ next of kin for publication of this case report, including accompanying images. One patient however had no immediate next of kin available for consent despite our attempts to make contact. Patient data has been anonymized to protect patient identity.
